# MicroRNA124-IL6R Mediates the Effect of Nicotine in Inflammatory Bowel Disease by Shifting Th1/Th2 Balance Toward Th1

**DOI:** 10.3389/fimmu.2020.00235

**Published:** 2020-02-21

**Authors:** Zhen Qin, Peng-Yuan Wang, Jing-Jing Wan, Yu Zhang, Jie Wei, Yang Sun, Xia Liu

**Affiliations:** ^1^Department of Clinical Pharmacy, School of Pharmacy, Second Military Medical University, Shanghai, China; ^2^Department of Pharmacology, School of Pharmacy, Second Military Medical University, Shanghai, China

**Keywords:** nicotine, Th1/Th2 balance, ulcerative colitis, Crohn's disease, miR-124, IL-6R

## Abstract

Epidemiological investigations have shown that smoking ameliorates ulcerative colitis (UC) but exacerbates Crohn's disease (CD), diseases that feature a Th2-mediated and Th1-mediated response, respectively. Cigarette extracts, especially nicotine, affect the Th1/Th2 balance. We previously reported that nicotine protects against mouse DSS colitis (similar to UC) by enhancing microRNA-124 (miR-124) expression. Intriguingly, elevation of miR-124 in CD is reported to aggravate the disease. Here we investigate the dual regulation of miR-124 in inflammatory bowel diseases (IBDs), which may explain the similar bidirectional regulation of tobacco. We found that overexpressed miR-124 protected against mouse DSS-induced colitis with a Th1 polarization in peripheral blood lymphocytes and colon tissues, which was also found in human peripheral blood lymphocytes. Conversely, miR-124 knockdown worsened DSS murine colitis with a Th2 polarization. Moreover, knockdown of miR-124 could eliminate the polarization toward Th1 after nicotine treatment, suggesting that miR-124 mediates the effect of nicotine on the Th1/Th2 balance. In addition, interference of IL-6R, which is a downstream target of miR-124, could remarkably weaken the Th1 polarization induced by miR-124. Taken together, these results suggest that nicotine shifts the balance of Th1/Th2 toward Th1 via a miR-124-mediated IL-6R pathway, which might explain its dual role in IBDs.

## Introduction

Inflammatory bowel diseases (IBDs), relapsing and chronic inflammatory gastrointestinal disorders, mainly consist of ulcerative colitis (UC) and Crohn's disease (CD). At present, the etiology and pathogenesis of IBDs have not been fully elucidated (1, 2). Environmental (3), genetic (4), and immunological factors (5) might form a complex interaction and contribute to IBD pathogenesis. UC and CD have apparent differences in their immunological features. UC is associated with a Th2-mediated response, characterized by increased levels of IL-4, IL-5, IL-6, and IL-13, whereas CD is related to a Th1-mediated response, with concomitant elevated production of IFN-γ, IL-2, and IL-18 (6).

A large number of epidemiological investigations have demonstrated that cigarette smoking is the strongest environmental factor associated with IBD. Smoking had a protective effect on UC patients, but the opposite effect on CD patients (7). Smokers had a lower UC risk, and decreased need for drug therapy and hospitalization. When compared with non-smokers, ex-smokers had a significantly elevated risk of UC (8). Conversely, smoking was reported to be an independent risk factor for CD progression. CD smokers had more severe disease courses with higher relapse rates, drug use, disease complications, and demand for surgery. Ex-smokers had less disease flare, immunosuppression, and steroid use than those who continued to smoke (9). Nicotine is a major component among the substances in cigarettes. Its administration, including nicotine-releasing enemas, gums, and patches, improves the symptoms and long-term prognosis in UC patients but not in CD patients (8). In mouse models, nicotine showed a protective role in oxazolone-induced colitis (UC-like Th2 cell-mediated colitis) but a deteriorative effect in TNBS-induced colitis (CD-like Th1 cell-mediated colitis) (10).

The balance between Th1 and Th2 is affected by smoking or nicotine. Cigarette extract alters dendritic cells function in CD patients, leading to Th1 polarization, while it increases Foxp3+ CD4 T cell prevalence in UC patients (11). In human lamina propria T cells, nicotine up-regulates T-bet (Th1 transcription factor) expression through α7nAChR, regulating intestinal immune balance to Th1 polarization and improving Th2 enteritis (12). In mice, the positive role of nicotine in oxazolone colitis has been related to increased colonic regulatory T cells and decreased Th17 cells and its aggravating effect in TNBS colitis to increased Th17 cells among colonic CD4 T cells (10). Recently, it was reported that tobacco extract directly acts on mouse CD4 T cells to promote Th1 polarization (13). These results suggest that nicotine might mediate the dual role of smoking on IBD by shifting the Th1/Th2 balance toward Th1 polarization.

However, the molecular mechanisms behind the dual role of nicotine on CD and UC remain elusive. We mentioned that nicotine is an agonist of α7nAChR in the cholinergic anti-inflammatory pathway. The intracellular mechanism that links α7nAChR activation and inflammation might provide valuable clues (14). MiR-124 has been demonstrated to be a crucial modulator of immunity and inflammation (15). In most cases, the inflammatory or immunological signals could induce miR-124 expression, which in turn functioned as a negative regulator to control inflammation (14–16). Our previous studies found that nicotine could specifically elevate the miR-124 level in macrophages through α7nAChR and exert anti-inflammatory effects in sepsis (14). Subsequent studies found that nicotine could protect against DSS colitis (resembling UC) by increasing the miR-124 level in colonic tissues and cells (17). Intriguingly, Zhao et al. also found miR-124 to be elevated in the intestinal epithelial cells and colonic tissues of active CD patients. However, they argued that miR-124 aggravated TNBS-induced experimental colitis, while miR-124 inhibitors could alleviate inflammation (18).

MiR-124 is elevated in samples of both UC and CD, while its effects in the two diseases are opposite. Considering that miR-124 could be specifically induced by nicotine, we speculated that miR-124 might mediate the dual effects of nicotine on UC and CD by regulating Th1 and Th2 balance. Here, we first clarified whether miR-124 regulated the Th1/Th2 balance and promoted Th1 polarization in DSS-induced colitis, then evaluated its role in nicotine-induced Th1/Th2 shifting. Finally, we explored its downstream target responsible for this shifting in order to give a mechanistic explanation for the bidirectional effect of nicotine in IBD.

## Materials and Methods

### Reagents

Nicotine was obtained from Sigma (St. Louis, USA), and DSS was from MPBIO (Santa Ana, USA). Locked nucleic acid (LNA)-miR-124 and its negative control were purchased from Exiqon (Woburn, USA). T-bet polyclonal antibody was obtained from ThermoFisher Scientific (Waltham, USA), GATA3 antibody was from Abcam (Cambridge, USA), and GAPDH antibody was from Protein Technology (Chicago, USA). BD™ Th1/Th2 cytokine CBA kit and Fixation/Permeabilization kit were purchased from BD Biosciences (San Jose, USA). IL-6 and IL-13 ELISA kits were obtained from Neobioscience (Shanghai, China). Recombinant human IL-2 was purchased from Novoprotein (Shanghai, China). PMA/Ionomycin and BFA/Monensin mixtures were from Multi Sciences (Hangzhou, China). Lymphocyte separation medium and human lymphocyte separation tubes were purchased from Dakewe (Shenzhen, China). MiR-124 agomir and IL-6R siRNA were from Ribobio (Guangzhou, China). Lipo6000 transfection reagent was purchased from Beyotime (Shanghai, China). Purified anti-CD3e and purified anti-CD28, fluorescence labeling flow cytometric mouse anti CD4-PerCP monoclonal antibody, mouse anti IFN-γ-PE monoclonal antibody, mouse anti IL-4-FITC monoclonal antibody, human anti CD4-FITC monoclonal antibody, human anti IFN-γ-PE (or APC) monoclonal antibody, and human anti IL-4-APC (or PE) monoclonal antibody were obtained from BioLegend (San Diego, USA).

### Animals and Treatment

Male C57BL/6 mice (8–10 weeks old) were provided by Sino-British SIPPR/BK Laboratory Animals, Shanghai, China. All experimental and surgical procedures were undertaken in accordance with the Scientific Investigation Board of the Second Military Medical University. Mice were given 3% DSS dissolved in filter-purified drinking water for up to 6–8 days to induce colitis. Detailed information on the administration of miR-124 agomir, LNA, or LNA together with nicotine is shown in the corresponding figure captions.

### Disease Activity Index (DAI) of DSS Colitis

The DAI scoring system described previously (17, 19) consists of body weight loss (zero, none; one, 1–5%; two, 5–10%; three, 10–15%; four, 15–20%), stool consistency (zero, normal; two, loose stools; four, watery diarrhea), and bloody stools (zero, normal; two, slight bleeding; four, gross bleeding). The sum of the three values constitutes the DAI.

### Hematoxylin and Eosin (H&E) Staining and Scoring

Histopathological colon sections were graded using the following system described previously (17): colonic mucosa thickness (zero to three), crypt abscess (zero to three), inflammatory cell infiltration (zero to three), and epithelial erosion (zero to three). The sum of the four values constitutes the HE score. Two technicians blinded to the sample sources scored all the slides independently, and the mean value is used as the valid score.

### Peripheral Blood Lymphocyte Isolation

Murine peripheral blood lymphocytes were isolated on the last day of DSS drinking. Human peripheral blood lymphocytes were collected from normal blood samples in Changhai Hospital, in accordance with the ethical standards of the institutional committee. These fresh blood samples were slowly added to a human lymphocyte separation tube, centrifuged at 800 g adjusted to three gears of acceleration (ACC) and decelerated (DEC) for 15 min. The middle level was carefully absorbed by dropper, washed twice and suspended in RPMI-1640 with 1 × 10^6^ cells/mL after discarding the supernatant.

### Human Peripheral Blood Lymphocyte Activation and Transfection

Isolated human lymphocytes were activated with IL-2 (80 ng/mL), anti-CD3e (50 ng/mL), and anti-CD28 (50 ng/mL) for 24 h (20). They were then transfected with miR-124 agomir (300 nM), LNA (200 ng/mL), or LNA together with nicotine (1 μM) separately (for detailed information, see the corresponding figure captions). Cells and supernatants were harvested, and related cytokines and proteins were detected by ELISA, CBA, flow cytometry, or Western blotting, separately.

### Flow Cytometry (FCM) Analysis

CD4+ T cells secreting IFN-γ and IL-4 were detected by flow cytometry (FCM), respectively representing Th1 and Th2 cells. Isolated peripheral blood lymphocytes (about 1–5 × 10^6^ cell volume) were suspended to 0.5 mL by RPMI-1640. Each tube was mixed with PMA/Ionomycin mixture and BFA/Monensin mixture, 2 μL each, followed by incubation at 37°C for 6 h. Then, 3 μL surface CD4 fluorescent antibody was added, and it was incubated in the dark for 30 min. Next, 300 μL Fixation/Permeabilization Kit with BD Golgi Stop™ was added, followed by incubation at 4°C for 30 min. Anti-IFN-γ fluorescent antibody or IL-4 fluorescent antibody at 2 μL each was added into the flow tube, and it was incubated at 4°C for 30 min. The results were acquired on a Becton Dickinson FACS Calibur flow cytometer (Frankin Lakes, USA) and analyzed using FlowJo (Tree Star Inc., Ashland, USA).

### Cytometric Bead Array (CBA) and ELISA

The culture supernatants of the human peripheral blood lymphocytes treated above were harvested. For murine colon tissues, homogenates were collected. Briefly, a total of 500 μL RIPA solution was added to every 100 mg of murine colon tissues; the cracking time on ice was more than 30 min. Tissues were further homogenized and centrifuged at 4°C, 13,000 g for 10 min, and the supernatants were harvested. Th1 factors IL-2, IFN-γ, and Th2 factors IL-4, IL-6, and IL-13 in colon tissue homogenates and cell culture supernatants were detected according to the instructions of the BD™ Th1/Th2 cytokine CBA Kit and the Neobioscience IL-6, IL-13 ELISA kit.

### Immunoblotting

The murine colon tissues and human peripheral blood lymphocytes were lysed, and proteins were separated using 10% SDS-PAGE and transferred onto nitrocellulose filter (NC) membranes. Immunoblotting for T-bet, GATA3, IL-6R, and GAPDH was conducted using specific antibodies. An Odyssey infrared fluorescence imaging system (Li-cor Bioscience, USA) was used to capture and analyze images.

### Immunohistochemical Staining

Murine colon tissue sections were blocked with 3% H_2_O_2_, 2% BSA in 0.01 M PBS (pH 7.3) for 30 min. After overnight incubation at 4°C with the anti-IL-6 antibody (1:300; rabbit, Abcam) or anti-CD4 antibody (1:1000; rabbit, Abcam), immunofluorescence of CD4 and IL-6 was performed using a Three-Color Fluorescence kit (Shanghai Recordbio Biological Technology, Shanghai, China) for 10–15 min at room temperature, based on the tyramide signal amplification (TSA) technology. After extensive washing, the nuclei were stained with DAPI. Negative controls were run concurrently, except that the antibody dilution buffer was used to substitute the primary antibody (21). They were scanned with a Leica SP5 (Mannheim, Germany) confocal laser scanning microscope equipped with a ×63 objective, and then with three times magnification. Three channels were used to acquire the images sequentially: FITC laser (488 nm, green for IL-6), Cy5 laser (633 nm, rose for CD4), and DAPI (405 nm, blue for nuclei). We captured images separately and then merged them. The cells showing co-localization of signals generated from both the green (IL-6) and rose (CD4) channels were identified as IL-6 + CD4 + T cells.

### Statistical Analysis

One-way analysis of variance and *post-hoc* Tukey's multiple comparison tests were performed when comparing multiple treatments. The Student's *t*-test was used for two groups with equal variance. In analyzing daily DAI scores and body weight change, repetitive measurement deviation analysis was conducted. A *P* < 0.05 was considered to be statistically significant.

## Results

### MiR-124 Overexpression Alleviates DSS Murine Colitis, Shifting the Th1/Th2 Balance Toward Th1 Polarization

**Figure 1:**
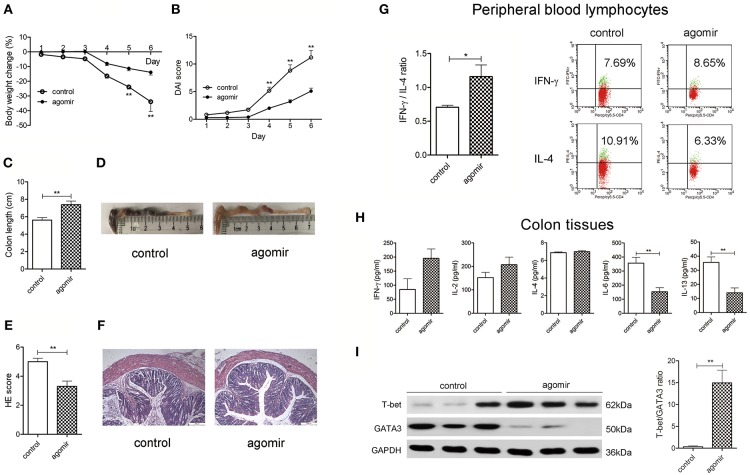
MiR-124 overexpression ameliorates DSS murine colitis, shifting the Th1/Th2 balance toward Th1 polarization. Mice were given 3% DSS in drinking water for 6 consecutive days. MiR-124 agomir (350 nmol/kg) or matched negative control was injected via the tail vein on 1 day ahead of DSS drinking and on the 4th day during DSS drinking. Body weight change (%) **(A)** and Disease Activity Index score (DAI, **B**) were analyzed by repetitive measurement deviation analysis and Student's *t*-test. Colon length **(C)** and HE score **(E)** were analyzed by Student's *t*-test. **(D,F)** Representative images of colon length and HE staining. *N* = 10–11 per group. **(G)** Peripheral blood lymphocytes from the above mice were treated with a PMA/Ionomycin and BFA/Monensin mixture for 6 h, then the Th1/Th2 (IFN-γ/IL-4) ratio was analyzed by FCM and Student's *t*-test (left). Representative flow image shows IFN-γ and IL-4 expression gated on CD4+ T cells (right). **(H)** Colon tissue homogenates were used to detect Th1-related cytokines IFN-γ and IL-2 and Th2-related cytokines IL-4, IL-6, and IL-13 by CBA and ELISA, and the data were analyzed by Student's *t*-test. *N* = 6–10 per group. **(I)** Representative Western blots (*N* = 3) of T-bet and GATA3 and corresponding quantification in colon tissues. All data represent mean ± SEM. ^*^p < 0.05, ^**^p < 0.01 vs. control group.

We first explored whether miR-124 could protect against DSS colitis in mice. Tail-vein injection of miR-124 agomir into DSS colitis mice overexpressed miR-124 (22). As shown in Figures 1A–F, compared to the control group (DSS-drinking with miR-124 agomir control), miR-124 overexpression induced a significant improvement in body weight loss, DAI score increase, colon length shortening, and HE score elevation, suggesting a protective role for miR-124 in DSS colitis.

Th1/Th2 ratio was further evaluated in isolated murine peripheral blood lymphocytes. As shown in Figure 1G, compared with the control group, miR-124 treatment increased the percentage of Th1 (IFN-γ labeling) in CD4+T cells, decreased that of Th2 (IL-4 labeling), and significantly increased the ratio of IFN-γ to IL-4. In murine inflammatory colon, we found an infiltration of CD4-positive T cells and Th2-related IL-6 expression. MiR-124 agomir administration decreased IL-6 immunofluorescence. Noteworthily, CD4-positive T cells were found to be co-localized with IL-6 (Supplementary Figure 1), indicating a Th2 phenotype. In order to further quantify the profile of Th1/Th2, the related cytokines in colon tissues were detected. MiR-124 dramatically decreased the level of Th2-related IL-6 and IL-13 in colonic tissues (Figure 1H). Transcription factors T-bet and GATA3 play a crucial role in promoting Th1 and Th2 cell development, respectively (23). We observed whether miR-124 affects their expression. MiR-124 agomir hugely increased the protein expression of T-bet and decreased that of GATA3 in colon tissues of DSS mice (Figure 1I), showing the effect of shifting the Th1/Th2 balance toward Th1 polarization.

### MiR-124 Knockdown Worsens DSS Murine Colitis, Shifting the Th1/Th2 Balance Toward Th2 Polarization

**Figure 2:**
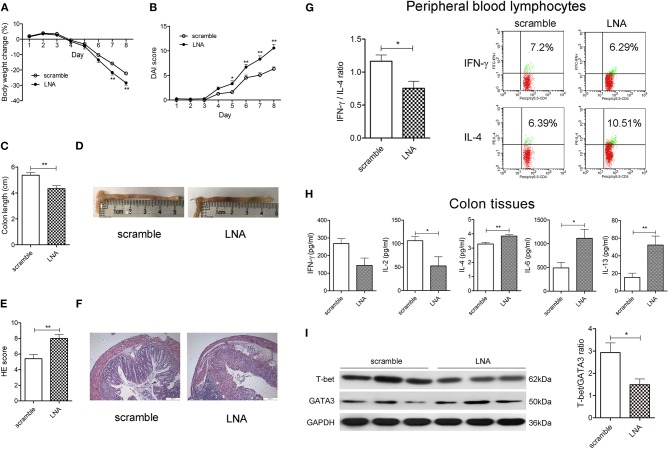
MiR-124 knockdown worsens DSS murine colitis, shifting the Th1/Th2 balance toward Th2 polarization. Mice were tail-vein injected daily with 10 mg/kg LNA-miR-124 or matched scramble control for 3 days. They were then given 3% DSS in drinking water for 8 consecutive days. Body weight change (%) **(A)**, DAI score **(B)**, colon length **(C)**, and HE score **(E)** were analyzed as mentioned in Figure 1. **(D,F)** Representative images of colon measurement and HE staining. *N* = 15–16 per group. **(G)** Th1/Th2 (IFN-γ/IL-4) ratio in peripheral blood lymphocytes was analyzed by FCM and Student's *t*-test, as mentioned in Figure 1. **(H)** Th1-related cytokines IFN-γ and IL-2 and Th2-related cytokines IL-4, IL-6, and IL-13 in colon tissue homogenates were detected by CBA and ELISA and analyzed by Student's *t*-test. *N* = 7–9 per group. **(I)** Representative Western blots (*N* = 3) of T-bet and GATA3 and corresponding quantification in colon tissues. All data represent mean ± SEM. ^*^p < 0.05, ^**^p < 0.01 vs. scramble group.

We then observed the effect of miR-124 knockdown on murine DSS colitis and the Th1/Th2 balance. Tail-vein injection of miR-124 LNA into DSS colitis mice knocked down miR-124 (17). As shown in Figures 2A–F, miR-124 knockdown led to a significant aggravation of weight loss, the DAI and HE scores, and colonic length as well as HE pathology. Meanwhile, it significantly decreased the ratio of IFN-γ to IL-4 in murine peripheral blood lymphocytes (Figure 2G). Also, it increased IL-6 immunofluorescence in colon tissues and its co-localization with CD4-positive cells (Supplementary Figure 2). Quantitative analysis showed that miR-124 knockdown significantly decreased Th1 cytokine IL-2 expression and increased Th2 cytokine IL-4, IL-6, and IL-13 expression in colonic tissues (Figure 2H). Furthermore, miR-124 LNA significantly decreased the protein expression of T-bet and increased that of GATA3 in colon tissues of DSS mice (Figure 2I), showing the effect of shifting the Th1/Th2 balance toward Th2 polarization.

### MiR-124 Promotes Th1 Polarization in Human Peripheral Blood Lymphocytes

**Figure 3:**
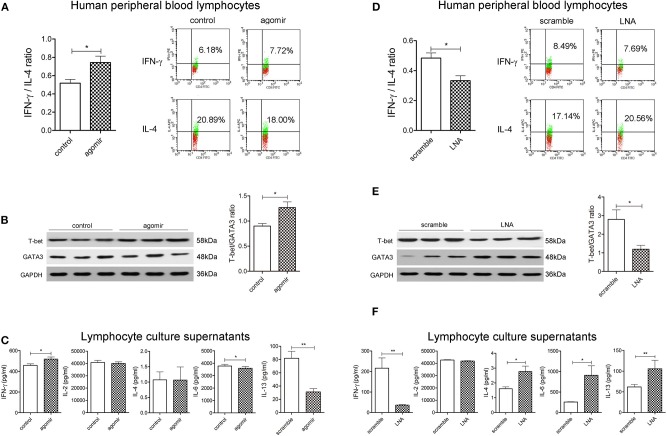
MiR-124 promotes Th1 polarization in human peripheral blood lymphocytes. Cells were treated with IL-2 (80 ng/mL), anti-CD3e (50 ng/mL), and anti-CD28 (50 ng/mL) for 24 h and transfected with agomir (300 nM) or miR-124 LNA (200 ng/mL) for 36 h. Before FCM detection, PMA/Ionomycin, and BFA/Monensin mixture were added for 6 h. **(A,D)** Th1/Th2 (IFN-γ/IL-4) ratio was analyzed and representative flow images gated on CD4+ T cells are shown. **(B,E)** Representative Western blots (N=3) of T-bet and GATA3 and corresponding quantification. **(C,F)** Th1-related cytokines IFN-γ and IL-2 and Th2-related cytokines IL-4, IL-6, and IL-13 in the supernatants were analyzed by CBA and ELISA. *N* = 6–9 per group. All data represent mean ± SEM. ^*^p < 0.05, ^**^p < 0.01 vs. control (or scramble) group by Student's *t*-test.

We further evaluated the role of miR-124 in the Th1/Th2 balance in human peripheral blood lymphocytes. After activation by IL-2, anti-CD3e, and anti-CD28, cells were transfected with miR-124 agomir for 36 h, and the percentage of Th1 (IFN-γ labeling) and Th2 (IL-4 labeling) in CD4+ T cells was detected by FCM. Results showed that miR-124 overexpression increased the percentage of Th1, decreased that of Th2, and significantly increased the ratio of IFN-γ and IL-4 (Figure 3A). MiR-124 agomir significantly increased the protein ratio of T-bet/GATA3 in human peripheral blood lymphocytes (Figure 3B). Accordingly, in their culture supernatants, the level of Th1 factor IFN-γ was dramatically increased, and the levels of Th2 factors IL-6 and IL-13 were significantly decreased (Figure 3C). MiR-124 knockdown by the LNA technique in human peripheral blood lymphocytes showed the opposite effect (Figures 3D–F). These data suggested that miR-124 could promote Th1 polarization in human peripheral blood lymphocytes.

### After miR-124 Was Knocked Down, the Promoting Effect of Nicotine on Th1 Polarization Disappeared

**Figure 4:**
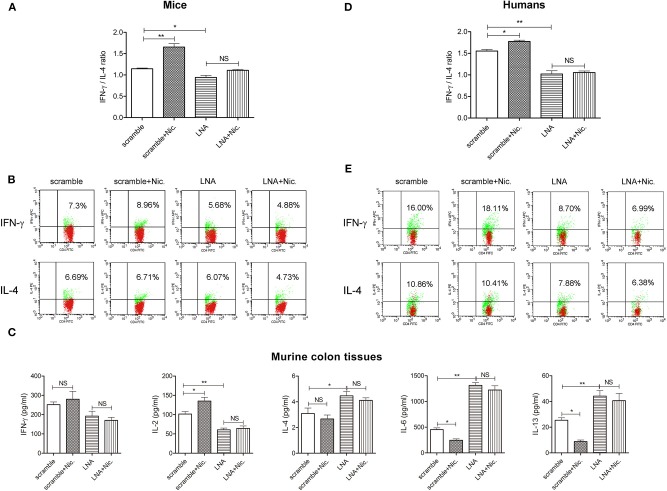
MiR-124 knockdown abolishes the role of nicotine in upregulating the Th1/Th2 ratio in murine and human peripheral blood lymphocytes. Mice were injected daily via the tail vein with 10 mg/kg of LNA-miR-124 or matched scramble control for 3 days. They were then given 3% DSS drinking water together with 0.3 mg/kg of nicotine (subcutaneous injection) for 7 consecutive days (*N* = 7–10). Human lymphocytes were isolated and treated with IL-2 (80 ng/mL), anti-CD3e (50 ng/mL), and anti-CD28 (50 ng/mL) for 24 h and then transfected with miR-124 LNA or corresponding scramble control (200 ng/mL) for 30 h, followed by with or without nicotine (1 μM) for 6 h. **(A,D)** Th1/Th2 (IFN-γ/IL-4) ratio was analyzed in each group. *N* = 3–6 per group. **(B,E)** Representative flow images showing IFN-γ and IL-4 expression gated on CD4+ T cells. **(C)** Th1-related cytokines IFN-γ and IL-2 and Th2-related cytokines IL-4, IL-6, and IL-13 in murine colon tissue homogenates were detected by CBA and ELISA. *N* = 8 per group. Data represent mean ± SEM and were analyzed by one-way ANOVA followed by *post-hoc* Tukey's test. ^*^p < 0.05, ^**^p < 0.01. NS, no significant.

We then observed the impact of nicotine on the Th1/Th2 ratio of murine DSS colitis and the influence of miR-124 knockdown on this process, in order to evaluate whether miR-124 mediates the regulatory effect of nicotine on Th1/Th2. Mice were injected daily via the tail vein with 10 mg/kg of LNA-miR-124 or a matched scramble control for 3 days. They were then given 3% DSS drinking water together with 0.3 mg/kg of nicotine (subcutaneous injection) for consecutive 7 days. Consistent with our previous report (17), nicotine ameliorated body weight loss, DAI score increase, colon length shortening, and HE score elevation. After miR-124 was knocked down, the protective effect of nicotine disappeared (Supplementary Figure 3). We further isolated mouse peripheral blood lymphocytes and detected the expression of Th1 factor IFN-γ and Th2 factor IL-4 by FCM. Compared with the scramble group, nicotine administration markedly increased the Th1/Th2 ratio. However, this role disappeared after miR-124 was knocked down (Figures 4A,B). Accordingly, nicotine administration dramatically increased Th1 cytokine IL-2 expression and decreased Th2 cytokine IL-6 or IL-13 expression in colon tissues. After miR-124 was knocked down, the effect of nicotine disappeared, indicating that nicotine shifted the Th1/Th2 balance through targeting miR-124 (Figure 4C). Similar results were also obtained in human peripheral blood lymphocytes (Figures 4D,E).

### After IL-6R Was Suppressed, the Promoting Effect of miR-124 on Th1 Polarization Disappeared

**Figure 5:**
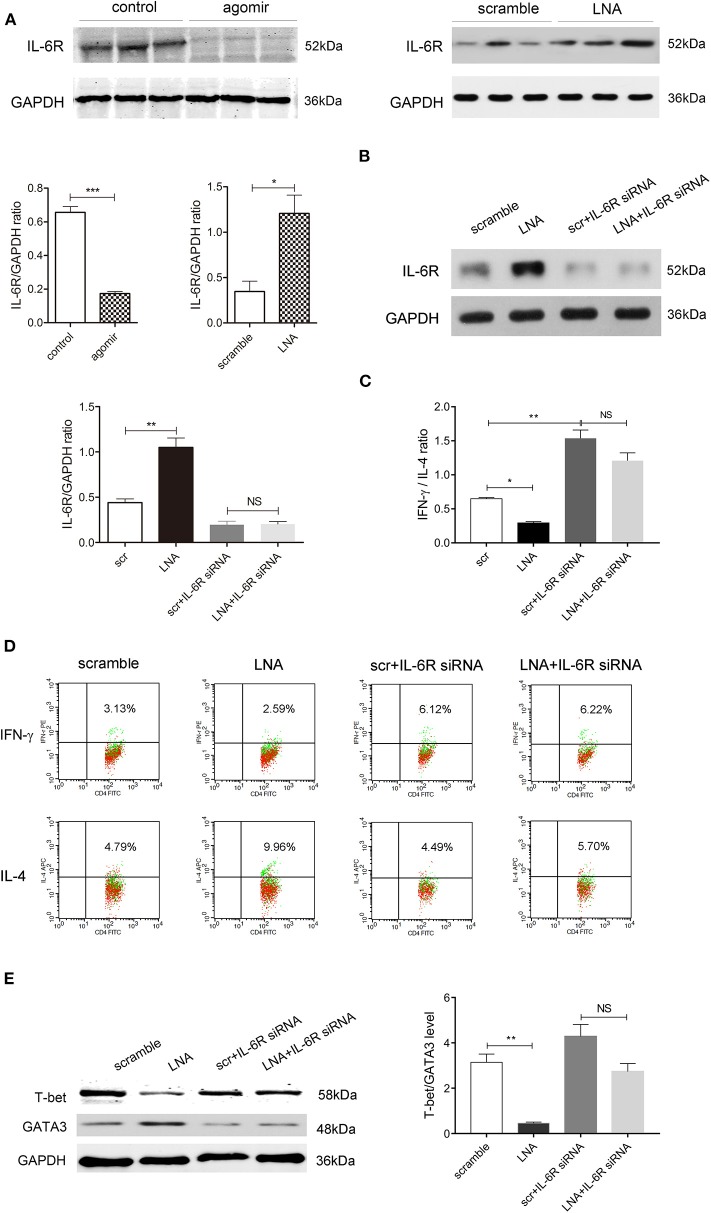
Inhibiting IL-6R abolishes the promoting effect of miR-124 on Th1 polarization. **(A)** The protein expression of IL-6R in colon tissues after miR-124 agomir or LNA administration in DSS mice, as mentioned in Figures 1, 2, was detected by Western blot (*N* = 3). Corresponding quantification is also shown. **(B–E)** Human lymphocytes were isolated and treated with IL-2 (80 ng/mL), anti-CD3e (50 ng/mL), and anti-CD28 (50 ng/mL) for 24 h and then transfected with miR-124 LNA or corresponding scramble control (200 ng/mL) for 60 h. They were then treated with or without IL-6R siRNA (100 nM) for 36 h. The protein expression of IL-6R and its corresponding quantification was analyzed by Western blot (*N* = 3) **(B)**. IFN-γ and IL-4 expression gated on CD4+ T cells were analyzed by FCM (*N* = 6 per group), and the IFN-γ/IL-4 ratio and representative flow images are shown in **(C,D)**. Representative Western blots (*N* = 3) of T-bet and GATA3 and corresponding quantification are shown in **(E)**. Data represent mean ± SEM and were analyzed by one-way ANOVA followed by *post-hoc* Tukey's test. ^*^p < 0.05, ^**^p < 0.01, ^***^p < 0.001. NS, no significant.

We then probed into the downstream target of miR-124 that regulates the Th1/Th2 balance. Many targets of miR-124 have been reported (24–26). Among them, IL-6R is reported to regulate the balance of Th1/Th2. High expression of IL-6Rα selectively produced Th2 factors IL-5 and IL-13, increased the GATA3 level, and decreased the T-bet level in CD8-positive human effective memory T cells of peripheral blood (27). Interestingly, miR-124 could target IL-6R, inhibit the activity of a luciferase reporter construct containing IL-6R 3'UTR, and consequently modulate the IL-6R/STAT3 pathway (24). We found that miR-124 agomir could significantly decrease while LNA could significantly increase the IL-6R level in colon tissues of DSS colitis mice (Figure 5A). We therefore speculated that IL-6R might mediate the effect of miR-124 in the Th1/Th2 balance.

Human peripheral blood lymphocytes were isolated, and IL-6R siRNA was transfected to suppress IL-6R protein expression (Figure 5B). FCM showed Th1 factor IFN-γ and Th2 factor IL-4 expression gated on CD4-positive cells. Compared with the scramble control group, miR-124 LNA administration markedly attenuated the ratio of IFN-γ to IL-4, which was abolished after IL-6R expression was suppressed. IL-6R siRNA alone significantly elevated IFN-γ/IL-4, mimicking the effect of miR-124 (Figures 5C,D). Accordingly, LNA significantly lowered the protein ratio of T-bet/GATA3. After IL-6R was knocked down, the effect of miR-124 LNA disappeared, indicating that miR-124 shifted the Th1/Th2 balance through targeting IL-6R (Figure 5E).

## Discussion

Aminosalicylic acid, corticosteroids, antibiotics, and immunosuppressive therapies are commonly used agents for the treatment of IBD. Because the specific mechanism remains unknown, therapy in many patients usually aims at non-specifically controlling inflammation (28). The discovery of new or specific therapies for IBD is urgently needed. Aberrant Th1 and Th2 responses contribute to IBD (29). UC reflects an excessive Th2 response, while CD is associated with aberrant Th1 response (30). More recent researches suggested that regulating the balance of Th1/Th2 might be a promising way to treat IBD.

Studies showed that agents that promote the Th2 profile or block Th1 response were beneficial in ameliorating CD symptoms (31, 32). Vasoactive intestinal peptide (VIP) had therapeutic and prophylactic effects in TNBS-induced colitis, which was related to the down-regulation role of the IFN-γ level in splenic and lamina propria CD4+ T cells (33). Schistosome egg diminished IFN-γ and enhanced IL-4 production from alpha CD3-stimulated mesenteric lymph node cells and spleen in TNBS-treated mice, thereby reducing colitis severity (34). Curcumin decreased the expression of Th1 cytokines (IL-12, IFN-γ, TNF-α) and increased that of Th2 cytokines (IL-4 and IL-10) in colon mucosa and thereby exerted therapeutic effects on TNBS-induced colitis (35).

Conversely, agents that skew toward the Th1 profile could lead to a reduction of UC disease activity (36). In peripheral CD4+ T cells, interferon or leukocytapheresis therapy in UC patients shifted the Th1/Th2 ratio to a Th1-dominant cytokine profile, which corresponded with an improvement in UC patients (37–39). Bifidobactrium longum could shift a Th1-dominant cytokine profile via T-bet upregulation, resulting in UC amelioration (40). Interestingly, infliximab, a chimeric monoclonal antibody directed against TNF-α, is effective both in patients with CD and with UC. In CD patients, IFX infusion was associated with an increase in Treg and a decrease in the ratio of Th1 (IFN-γ) to Th2 (IL-4) (41). In UC patients, blockade of TNF-α with infliximab increased the Th1 (IFN-γ)/Th2 (IL-4) ratio of peripheral CD4 T cells, leading to reduced disease activity (36, 42).

The experimental model of DSS murine colitis has provided valuable insight into the role of Th subsets and their secreted cytokines (43, 44). Our present studies first demonstrate that miR-124 protects against DSS colitis and shifts the Th1/Th2 balance toward Th1 polarization. Consistent with our findings, Wei et al. found that miR-124 overexpression in naive T cells inhibited the induction of FoxP3+Treg and enhanced the differentiation of IFN-γ+Th1 cells, therefore contributing to T cell-mediated immune clearance of glioma (45, 46). The regulation of Th1/Th2 by miR-124 was consistent with the Th1 promotion effect of nicotine in CD samples (10). We further found that miR-124 knockdown could abolish the Th1 polarization effect of nicotine on DSS mice and human peripheral blood lymphocytes. These data provided a reasonable explanation of the dual role of nicotine in IBD. That is, nicotine specifically elevates the miR-124 level in both UC and CD, and the latter promotes Th1 polarization, therefore exerting a protective effect in Th2-type UC and a deteriorative effect in Th1-type CD.

According to the previous studies, IL-6R was chosen as the candidate target of miR-124 in Th1 polarization (27). MiR-124 agomir administration inhibited IL-6R expression, while LNA administration augmented IL-6R expression in the colon tissues of DSS mice. In human peripheral blood lymphocytes, miR-124 knockdown decreased the IFN-γ/IL-4 ratio, which was abolished when IL-6R was suppressed. Moreover, IL-6R siRNA alone significantly elevated the ratio of IFN-γ to IL-4, mimicking the effect of miR-124. Therefore, it is conceivable that IL-6R might be the possible target mediating Th1/Th2 regulation by miR-124 under the microenvironment of CD or UC.

The IL6-IL6R-STAT3 signaling axis is crucial for inducing lineage-specific transcription factor. Soluble IL-6 binds with high affinity to the IL-6Rα chain and signal-transducing β-subunit gp130, inducing homodimerization of gp130. Activation of gp130 homodimer leads to tyrosine kinase phosphorylation of the JAK family, as well as the recruitment and activation of STAT-1 and STAT-3 (47–54). STAT3 plays an important role in the pathogenesis of UC (55–57). The p-STAT3 level is directly related to the extent of inflammatory pathology in colonic tissues (55). Our previous report found that blocking STAT3 activity was beneficial for murine DSS colitis and also abolished nicotine's protective effect (17). In another study, specific STAT3 deletion caused mouse death at 4–6 weeks old with CD-like pathogenesis and over-produced inflammatory cytokines, including TNF-α and IFN-γ (58, 59). Although there are some reports that STAT3 is necessary for Th1 and Th17 cell differentiation and development in CNS autoimmunity and collagen-induced arthritis (60–63), more data have revealed that STAT3 deficiency impaired RORγt expression (the transcription factor of Th17) and led to elevated T-bet expression (the transcription factor of Th1) expressed in T cells and Foxp3 (64). Furthermore, IL-6-treated pDCs enhanced the IL-4 mRNA level in naive allogeneic CD4+ T cells, favoring the Th2 over the Th1 type of response (65). MiR-124 is confirmed to directly target STAT3 in many cell types, including T cells (46, 66). We speculated that miR-124 might target the IL6-IL6R-STAT3 axis, forming a positive loop to promote Th1 polarization.

## Conclusions

**Figure 6:**
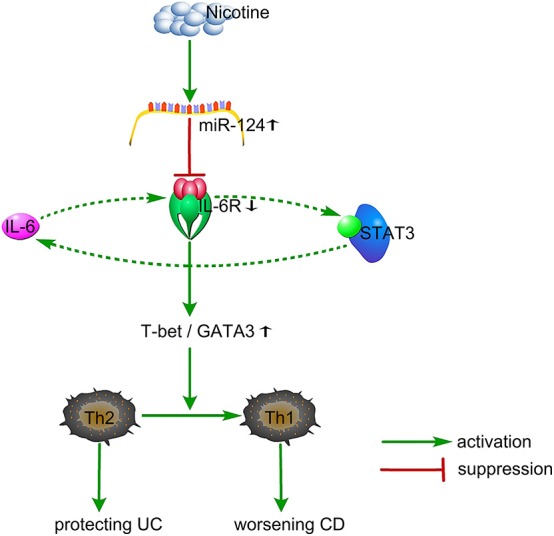
Proposed working model. Nicotine specifically elevates the miR-124 level. MiR-124 targets and down-regulates IL6R, resulting in an increased ratio of T-bet/GATA3 and a shifting from Th2 toward Th1, thereby protecting against Th2-type UC and worsening Th1-type CD. Additionally, IL6-IL6R-STAT3 might form a positive loop to regulate this process. UC, ulcerative colitis; CD, Crohn's disease.

Nicotine shifts the Th1/Th2 balance toward Th1 through the miR-124-IL-6R pathway, which might be responsible for its dual role in IBD (Figure 6). MiR-124 might act as a common target for the differential treatment of IBD, with up-regulation for UC patients and down-regulation for CD patients.

## Data Availability Statement

All datasets generated for this study are included in the article/Supplementary Material.

## Ethics Statement

The studies involving human participants were reviewed and approved by the Ethical Committee of Changhai Hospital. The patients/participants provided their written informed consent to participate in this study. The animal study was reviewed and approved by the Scientific Investigation Board of Second Military Medical University.

## Author Contributions

ZQ and XL designed the study. ZQ drafted the article. XL supervised the experiment and revised the manuscript. YS, P-YW, J-JW, and YZ provided help in animal model preparation and sample collection. JW and YS analyzed data. All authors approved the final version to be submitted.

### Conflict of Interest

The authors declare that the research was conducted in the absence of any commercial or financial relationships that could be construed as a potential conflict of interest.

## Abbreviations

UC: ulcerative colitis
CD: Crohn's disease
IBD: inflammatory bowel disease
LNA: locked nucleic acid
DAI: disease activity index
HE: Hematoxylin-eosin
FCM: flow cytometry
CBA: cytometric bead array.
