# Supplementary material for: MicroRNA124-IL6R Mediates the Effect of Nicotine in Inflammatory Bowel Disease by Shifting Th1/Th2 Balance Toward Th1
Source: Front Immunol. 2020 Feb 21;11:235. doi: 10.3389/fimmu.2020.00235 (PMC7050625; doi:10.3389/fimmu.2020.00235)
Supplement: Supplementary file 1 [file Data_Sheet_1.docx]

**
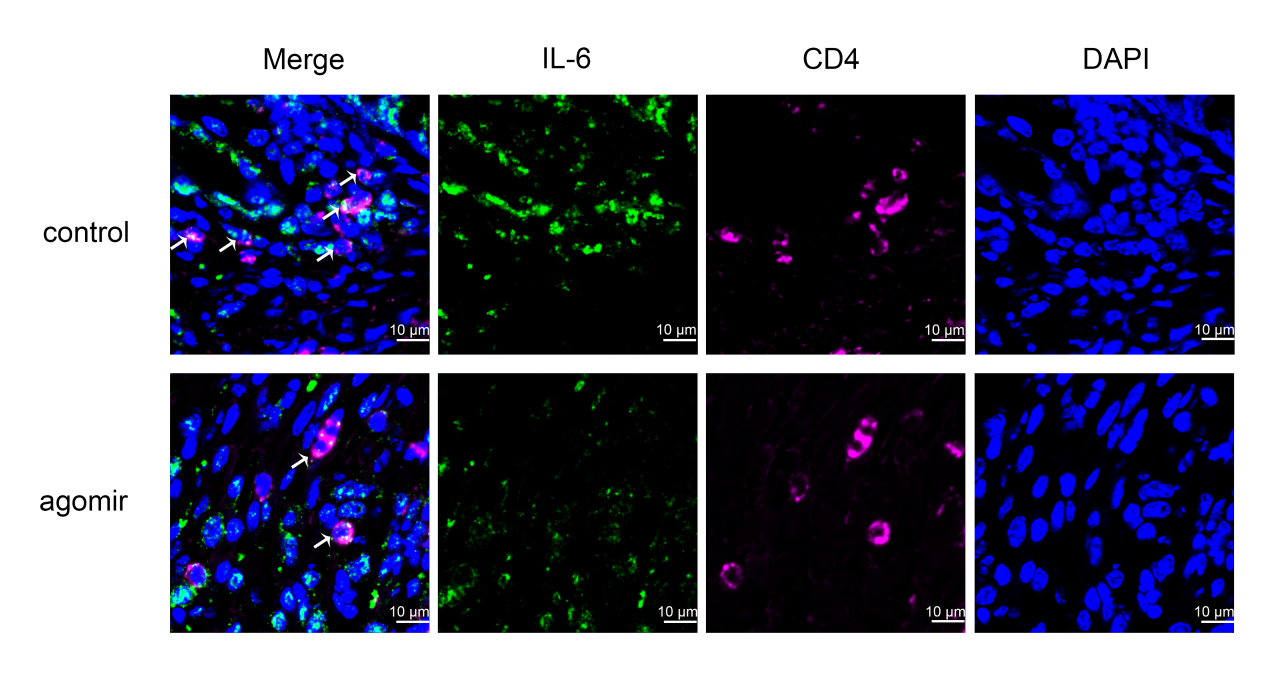
Supplementary Figure 1.** The effect of miR-124 agomir on the infiltration of CD4 positive T cells and IL-6 level in colon tissue. Mice were treated as Figure.1. Colon tissue section was performed immunofluorescence histochemistry of CD4 and IL-6. Nuclei were stained with DAPI. Panels from left to right: Merged; IL-6, green; CD4 positive T cells, rose; DAPI, blue. White arrows denote co-localization of IL-6 (green) and CD4+ T cells (rose). Original magnification 189×; Bar = 10 µm


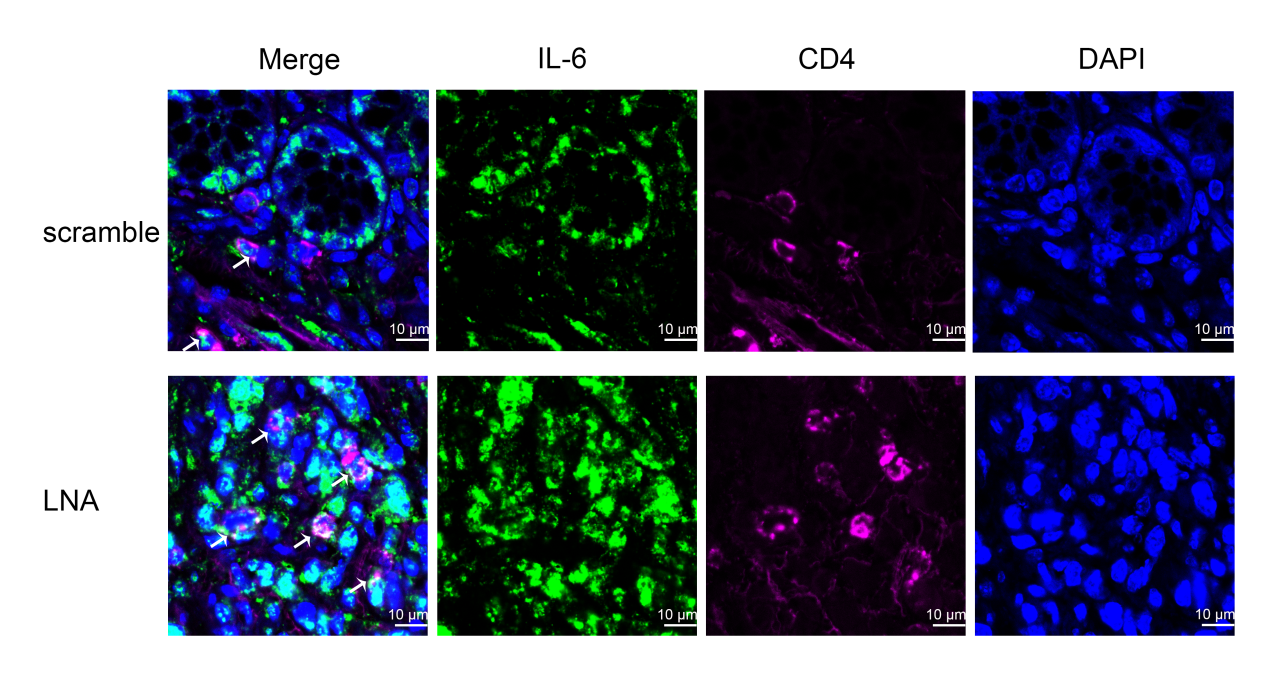


**Supplementary Figure 2.** The effect of miR-124 LNA on the infiltration of CD4 positive T cells and IL-6 level in colon tissue. Mice were treated as Figure.2. Colon tissue section was performed immunofluorescence histochemistry of CD4 and IL-6. Nuclei were stained with DAPI. Panels from left to right: Merged; IL-6, green; CD4 positive T cells, rose; DAPI, blue. White arrows denote co-localization of IL-6 (green) and CD4+ T cells (rose). Original magnification 189×; Bar = 10 µm

**
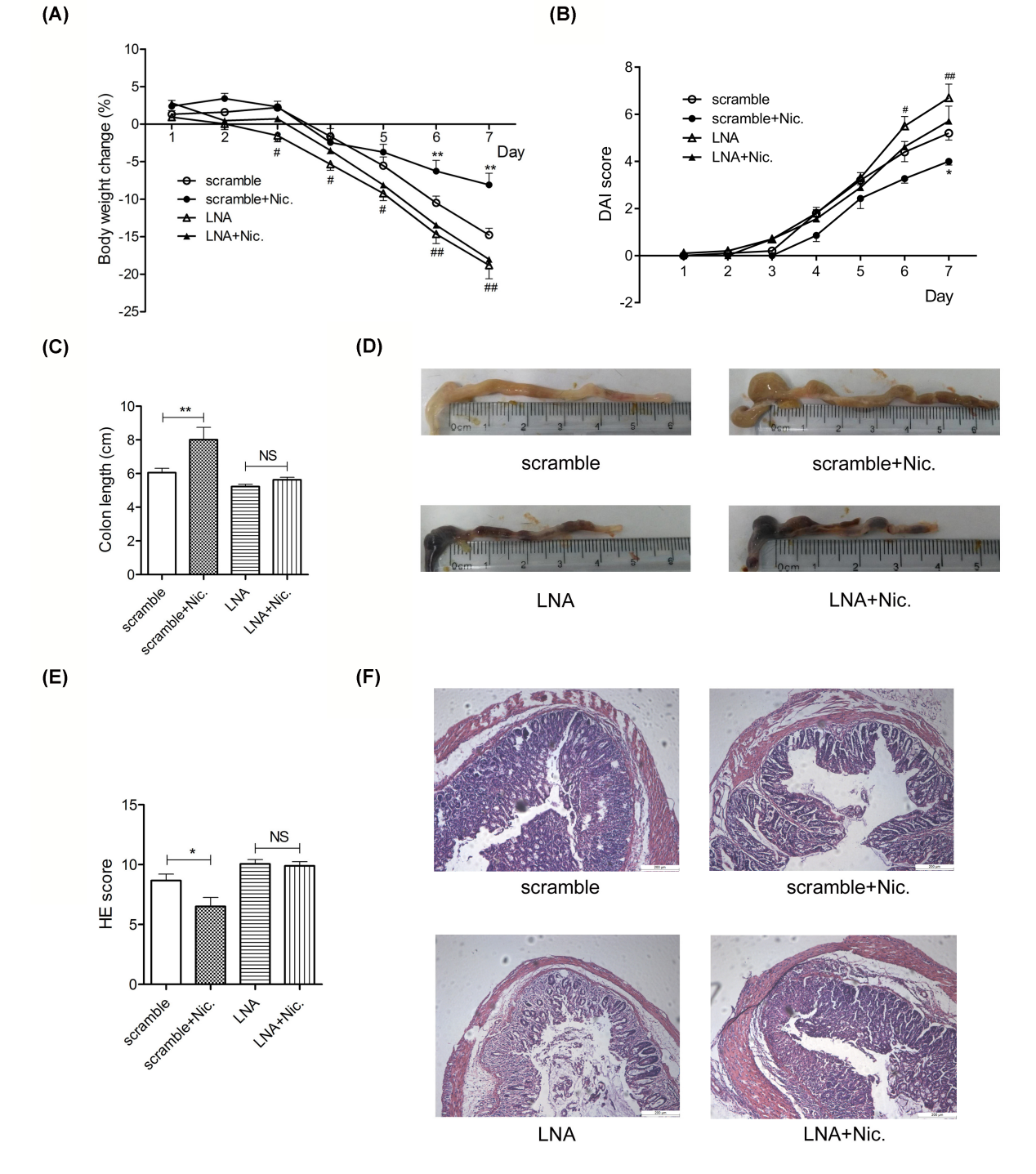
Supplementary Figure 3.** MiR-124 mediates the protective role of nicotine in murine DSS colitis. Mice were daily tail-vein injected with 10 mg/kg of LNA-miR-124 or matched scramble control for 3 days. They were then given 3% DSS in drinking water and subcutaneous injection of nicotine (0.3 mg/kg) for consecutive 7 days. Body weight change (%) (**A**) and Disease activity index score (DAI, **B**) were analyzed by repetitive measurement deviation analysis and Bonferroni post hoc tests. Colon length (**C**) and HE score (**E**) were analyzed by one-way ANOVA followed by Tukey’s post-hoc test. (**D, F**) Representative images of colon measurement and HE staining. N=7-10 per group. All data represent means ± SEM. **p* < 0.05, ***p* < 0.01 scramble + Nic. versus scramble group; #*p*<0.05, ##*p*<0.01 LNA versus scramble group.
